# Perceiving violence against healthcare workers in a child and adolescent emergency psychiatric ward in Hungary: a qualitative pilot study

**DOI:** 10.3389/fpsyg.2023.1220183

**Published:** 2023-11-01

**Authors:** Gyula Sófi, Krisztina Törő, Gábor Csikós, Gergely Fliegauf

**Affiliations:** ^1^Child and Adolescent Psychiatric Unit, Heim Pál National Pediatric Institute, Budapest, Hungary; ^2^Department of Personality and Clinical Psychology, Institute of Psychology, Pázmány Péter Catholic University, Budapest, Hungary; ^3^András Pető Faculty of Semmelweis University, Budapest, Hungary; ^4^National University of Public Service, Budapest, Hungary

**Keywords:** multi-causal model, doctor-patient relationship, high-security psychiatry, workplace violence, relatives in psychiatry, debriefing, child and adolescent psychiatry

## Abstract

**Aim:**

Psychiatry is a challenging setting that requires extraordinary effort from the staff. Healthcare workers in the field of psychiatry face substantial levels of violence, making the identification of abuse risk factors a social concern. Both the conduct of the children and their relatives can pose potential harm. Our study delved into the criminological and psychiatric factors underlying violence against healthcare workers.

**Methodology:**

We used qualitative, semi-structured, self-developed, online questionnaire involving 21 respondents. The participants were representing the staff composition of our department. The data set was coded in two phases using a multi-stage content analysis method. The results were compared with Hungarian and international literature.

**Findings:**

Among the participants, 52% reported no instances of physical abuse. The most prevalent form of perceived non-contact abuse was threats, accounting for 38% of reported cases. The identified risk factors for abuse included the child’s psychiatric disorder, communication issues, parental behavior, and low socio-economic status. Psychological trauma was identified as the most severe consequence. The respondents’ opinion indicated that the most common cause of violence (52%) was attributed to the child’s mental disorder. Workers primarily deal with abuse through negative emotions, with 76% of them reporting feelings of victimization. Additionally, 43% believed that abuse cannot be avoided, while 19% emphasized the significance of worker competence.

**Value:**

Our research can help to identify risk factors in child psychiatry wards and provide guidance for developing effective responses to violence against healthcare workers in Hungary, especially at our ward.

## Introduction

Violence against healthcare workers (VAHCW) encompasses both physical and psychological forms of aggression experienced by healthcare professionals in their work environment. The motivation behind such violence can stem from both criminological and psychiatric factors.

In Hungary, such acts can be considered as criminal offenses. The media pays special attention to these cases, though healthcare workers rarely report them. A recent Hungarian study (Ráczkevy-Deák and Besenyő, 2022) revealed a high level of latency related to verbal insults, 45% and physical violence, 52%. Another research (Irinyi et al., 2017) concluded that only 4.4% of healthcare workers do not experience violence during their work.

Risk factors of VAHCW can be divided into three groups: patient-related factors, external factors, and situational factors. A meta-analysis by MohammadiGorji et al. (2021) explored internal, external, and situational motives behind VAHCW, with internal factors pertaining to patients, external factors to the environment, and the third factor linked to low-quality staff-patient relationships. Angland et al. (2014) explored the perceptions of nurses. Among the patient-related factors, drug and alcohol abuse turned out to be crucial. External causes included a shortage of nursing staff, inadequate security measures, overcrowding, night shifts, and insufficient life-work balance. The inexperience of staff, communication problems, staff attitudes, and the demanding behavior of patients was listed among the situational factors. Similarly, Brophy et al. (2017) identified physical, psychological, interpersonal, and financial motives. They found environmental, organizational, social, and material risk factors. In their ethnographically inspired research of Lau et al. (2012) highlighted situational risk factors like the first few hours, long waiting, the social representation of the staff and the patient, and victim blaming. They also described less authoritative settings as more protective.

Earlier results (Pich et al., 2011) showed that long waiting times, lack of aggression management training and debriefing, and patronizing communication of the staff were among the motives. Internal factors included mental disorders, low socio-economic status (SES), and alcohol or drug abuse. Older and experienced staff play a protective role. However, a quantitative study with children diagnosed with schizophrenia (Goethals and van Marle, 2007) explained aggressive behavior with more patient-related features. They revealed schizophrenic parents, history of offending, antisocial behavior, and attention problems also as risk factors. A meta-analysis of 428 scientific articles (Dack et al., 2013) linked the patient-related risk factors in young men: emergency admission, loneliness, schizophrenia diagnosis, previous inpatient psychiatric treatment, history of violence and self-harm, and drug use. For girls, only substance use and previous history of violence counted.

Risk factors are also shaped by attribution bias (Ezeobele et al., 2019), healthcare staff tends to blame the patients first. Even higher qualified nurses regard the patients as the source of risk and not the external or situational factors. According to the decision-making model of Moylan and Cullinan (2011), three factors influence staff responses: (1) options (e.g., supportive intervention, medication, and restraint), (2) perceived information, and (3) the acceptance of aggression. Tolerance of violence, formal and informal education, individual acceptance of restraint, and professional standards are also relevant issues. It is challenging to harmonize professional standards and emotional reactions in some situations. In child psychiatric institutions, staff is lacking a well-defined view on aggression and on interventions (Fleury and Van Engelen, 2007).

Several qualitative studies (Bonner et al., 2002; Iozzino et al., 2015) underlined that physical and mechanical restraint can be traumatic for staff and patients. Additional risk factors are progressive mental disorder, refusal to take medication, and substance use. Medical staff can also commit violent acts, which are considered ill-treatment from a human rights perspective. According to a German study of more than 2,000 people (Hoffmann et al., 2020), one-third of former patients in child and adolescent psychiatric hospitals reported that they had been abused in some way by nurses. The majority of them were physically ill-treated (31.7%). From the research, it seems that boys who were more likely to be physically abused, while girls were subjected to excessive restraint. Emotional abuse was experienced by 23.1% of the respondents. Humiliation, insults, threats, or intimidation were also alleged.

Early traumatization is an underlying factor of psychiatric disorders. Childhood trauma (physical, psychological, or sexual abuse) is associated with neuropsychiatric consequences, and a large scale of mental disorders: depression, dissociative symptoms, opposition, dysthymia, obsessive-compulsive traits, phobias, anxiety, post-traumatic stress disorder, drug abuse, borderline personality disorder, attention deficit hyperactivity disorder, as well as schizophrenia (Gaskill and Perry, 2012). Aggression can be a manifestation of all these symptoms. The brain developing in a trauma-environment mobilizes maladaptive functions. These mechanisms serve to survive in a situation in which analytical thinking would be more damaging (Perry, 2008). Childhood abuse also changes behavior patterns (Anda et al., 2006). The fight-or-flight reactions of children exposed to this type of stressor are uncontrollable and unpredictable. Pathology is therefore an indirect factor of VAHCW, the problem is rooted in adverse childhood experiences. Since any child abuse is a criminal act and violence perpetrated by adolescents is dangerous to society, the criminological factors cannot be overlooked (Perry, 1984).

Conversely, aggressive behavior exhibited by either the child or their relatives adversely impacts the self-esteem and dignity of the staff, potentially resulting in burnout. Workplace hazards (Gale et al., 2009) can take several forms: sexual harassment, threats to family, damage to property, and stalking.

Adverse consequences of VAHCW include leaving the workplace, anger, helplessness, isolation, negative attitudes toward the employer, psychosomatic symptoms, reduced work performance, and post-traumatic stress disorder (PTSD) (Gillespie et al., 2013). Alden et al. (2008) compared the involvement of witnesses and participating individuals. They concluded that the affected people experienced greater fear during VAHCW, had more arousal symptom complexes (PTSD E), and they were more dissatisfied with their jobs. Witnesses also exhibited PTSD-like symptoms but interpreted them as a weakness in their personality (PTSD D). Patient aggression causes anxiety or anger in staff (Nijman, 2002), which creates communication problems. When combined with environmental factors, this can give rise to a detrimental cycle of violence.

In light of these findings, it is recommended to scrutinize the risk factors in a child and adolescent psychiatric unit in Hungary, as no targeted research has been conducted so far. The key question is the Hungarian characteristics and their uniqueness compared to international research. In this paper, we examine how healthcare staff experience and process violence at work and what are their most frequently expressed thoughts on the subject.

## Methods

In this study, we use the term “risk factor” in the sense of circumstances and preliminary signs staff considers important in the development of VAHCW. The multiple coding and content analysis was based on the methodology of three articles (Burnard, 1991; Braun and Clarke, 2006; Bengtsson, 2016). As a result, trends—a frequency hierarchy of items—were identified along the questions.

The inclusion criterion for the sample required individuals to possess prior a total of 1 year experience within both medical and psychiatric wards. Nevertheless, a unique exception was granted to an individual, a general practitioner with a completed 6 years long medical faculty program, who had recently initiated their residency within the department, despite not meeting the initial criteria. We believed their fresh insights would also contribute to our knowledge.

At the time of data collection, the department had a staff complement of 40 individuals. Additionally, we extended our outreach to employees who had been employed within the department in the year preceding the survey but had subsequently departed for various reasons. In totality, we made contact with 51 individuals, and 21 (41.18%) of these respondents completed the questionnaire. We aimed to invite subjects from diverse occupational backgrounds, including but not limited to medical professionals such as doctors and nurses, within our sample. Our rationale for achieving representativeness was predicated on securing a minimum of 20 respondents for the questionnaire. Increasing the number of participants could have posed a risk of pushing their involvement in the research. The data collection period was planned to last approximately 4 weeks, from the 11th of October to the 11th of November 2022. In the research field, previous qualitative studies have also employed relatively small sample sizes (Bonner et al., 2002; Pich et al., 2011; Angland et al., 2014).

The research took place at the Child and Adolescent Psychiatry Unit of Heim Pál National Pediatric Institute (Hospital). Considering the sensitivity of the topic and the nature of the work, audio-recorded interviews were not feasible. Therefore, an online qualitative questionnaire was developed, which underwent four modifications following consultations with experts, including Hungarian researchers in the field and professionals from the Unit. Discussions on the questionnaire started at the end of September 2022. The Head of the Institute authorized the research in October 2022. Data protection and privacy statements were prepared.

The questionnaire covered 10 issues: definition of contact and non-contact violence, case descriptions, perceived risk factors, consequences, triggers, processing, reporting, prevention, and other comments.

Elaborative explanations were required. The questionnaires were sent out in several phases by email or via social network messages to employees in the department.

## Results

The online data collection method ensured anonymity and considerably increased confidence in the research. We have consolidated the sociodemographic data of the 21 participants into a unified chart (Table 1).

**Table 1:** Participant demographics derived from content analysis of narrative responses to survey questions.

Features	*n* (21)	%
Gender		
Female	15	71
Male	6	29
Average age (year/minimum/maximum)	39,6/23/55	
Marital status		
In a relationship	8	38
Married	6	28
Single	5	24
Divorced	2	10
Education		
University	7	33
Technical and further education	7	33
High school	4	19
Bachelor degree in progress	2	10
PhD	1	5
Medical training level		
Physician (of which child and adolescent psychiatrist)	5 (1)	24 (5)
Nurse	5	24
Specialist nurse	5	24
Other^*^	3	14
No medical qualification^**^	3	14
Position		
Manager	0	0
Employee	21	100
Work schedule		
12-h shift	8	38
Constant daytime shift (8 h; 7 am to 3 pm)	7	33
8-h on working days shift (8 am to 4 pm)	5	24
24/48-h shift	1	5
Total years participants spent in health care/average/minimum/maximum	233/11,1/0/34	
Total years participants spent in the department/average/minimum/maximum	58/2,76/0/14	
Monthly overtime in hours total/average/minimum/maximum	256,5/12,21/0/70	

^*^Graduates with Master’s degrees. Further specifics cannot be disclosed to safeguard against potential identification.^**^Individuals with a secondary school education. Further specifics cannot be disclosed to safeguard against potential identification.

The total number of characters in the corpus was 35,057. A total of 412 extracts were subjected to multilevel coding and frequency ranking based on the responses, as detailed in Table 2. Subsequently, these extract types were condensed into 133 distinct items. In our analytical approach, we focused on the three most frequently occurring content items for each question.

**Table 2:** Overview of online self-completion questionnaire responses post-content analysis.

Questions and three most common items	n^*^	%^**^
Perception of physical violence		
It causes physical harm	13	62
Rooted in the mental disorders of children	6	29
Forms: punch, kick, bite, push, push down, and spit	6	29
Non-contact assault		
Degrading	18	86
Threat	8	38
Bullying (mocking, insulting, blackmailing, and blaming)	5	24
Content of case descriptions		
Reported no VAHCW	11	52
Narcissistic traits in parents (entitlement, devaluation, sense of importance, arrogance, and lack of empathy)	8	38
Attack with a chair	6	29
Perception of risk factors***		
Psychiatric disorder of the child	3	14
Communication between staff and patients	3	14
Emotions of the parents (worry, frustration, and hopelessness)	3	14
Low socioeconomic status of the family	3	14
Consequences		
Psychological harm	8	38
Trivialization of the consequences	7	33
Physical harm	6	29
Triggers		
The psychiatric disorder of the child	11	52
Negative emotions (aggression, hopelessness, and despair)	8	38
Maladaptation of the child (socialization, recognition of illness, lack of information, and inadequate coping)	6	29
Managing and processing VAHCW		
Negative emotional reactions	12	57
Self-reflection	9	43
Tried to handle the situation verbally	6	29
Reporting		
The information is transmitted	16	76
Reporting the management	11	52
Instant report	6	29
Prevention		
VAHCW cannot be prevented.	9	43
Training and workshops should be organized to improve communication, value orientation, and emotion regulation.	8	38
Professional protocols should be established.	4	19
Other comments		
Employee aptitude	4	19
Communication is important	2	10
Debriefing needed	2	10

^*^n, Number of times the item appeared in the responses;^**^%, compared to 21 responses;^***^The four most frequent responses had the same value.

Almost all the phenomena mentioned in the introduction are confirmed by our results. The most common forms for assault (hitting, kicking, biting, pushing, pushing down, and spitting) is attributed to the child’s mental disorder, and the perceived consequence is physical injury. This implies that the staff of the Unit consider children’s aggressive acts as a symptom of their disorder, in line with the results of previous research (Goethals and van Marle, 2007; Gaskill and Perry, 2012; Dack et al., 2013). At the same time, the responses also pointed to situational and external factors, as expected in light of other previous research (Brophy et al., 2017; MohammadiGorji et al., 2021). Non-physical abuse included humiliation and threats, which are primarily expressed by parents, but also by children toward the staff; these acts were explained by interpersonal factors in our research (see also Brophy et al., 2017). Our findings suggest that the self-esteem and dignity of the staff are often compromised and that bullying can occur not only in face-to-face interactions but also via email or telephone. In contrast to a previous findings (Irinyi et al., 2017), the majority of respondents (52%) in this research stated that they had not experienced violence. This result can be explained by the perception of children by the staff members, who see these children as sick persons who need help, and therefore their aggression is not regarded as abuse. In addition, staff identified patterns of abusive behavior on the parents’ side that could be described as narcissistic (entitlement, devaluation, sense of importance, arrogance, and lack of empathy) and identified as forms of verbal abuse. Assault with a chair was a frequently flagged issue.

Risk factors included psychiatric disorder of the child, communication, parental emotions, and low SES in the family, as predicted by other research (Pich et al., 2011). Psychological trauma, trivialization, physical trauma, and utilizing de-escalation techniques were among the consequences. This suggests that psychological injury has a greater impact on workers than physical injury or the threat of physical injury. The question on causes revealed the child’s illness, negative emotions, and other deficits in the child’s personality development. Employees see multiple and interrelated components behind the violence. It appears that staff, in line with previous research (Alden et al., 2008; Gillespie et al., 2013), mostly deal with the situation with negative emotional reactions, but at the same time they are able to self-reflect and prevent more serious harm.

In our sample, the majority, 76% of workers, immediately pass on information about bullying to their managers. This proportion is higher than a Hungarian trend described recently (Ráczkevy-Deák and Besenyő, 2022). It is believed that bullying cannot be prevented, but training could be organized and protocols should be developed. This implies that workers could have a possible solution to combat abuse despite feeling powerless. In line with the previous research (Meerwijk et al., 2007) we found, it is important that those working directly with patients have to be competent in psychiatric work, communicate well with children, and that debriefing sessions should take place after abuse.

In this research, risk factors were reported related to the visitation, admission, mechanical restraint, and to the withdrawal symptoms. This supports previous research findings (Bonner et al., 2002; Iozzino et al., 2015; Hoffmann et al., 2020). Patient admission was considered as consequences and causes too. This may be explained by the fact that both the child and their parents are confronted during the patient’s admission with the fact that the patient will spend more time in the ward. While waiting for an admitting medical doctor and the examination, the child is most likely to leave the institution. The parent perceives the child’s resistance, and on the other hand, the doctor is obliged to withdraw the patient’s liberty due to emergency psychiatric symptoms. In such cases, scenarios can be escalated, which in our research were classified as situation conflicts (Lau et al., 2012). The three problematic cornerstones of the admission are the announcement of the decision, the deprivation measures to be taken (e.g., taking the telephone and the tobacco product), and the moment when the child leaves the relatives. In practice, several minutes may elapse between these three moments. Conflict escalations leading to physical restraint are often resulting from avoid self-harm, as several studies have indicated (Bonner et al., 2002; Iozzino et al., 2015). Among other contributors, staff burnout and anxiety, unpredictable, abused and fleeing children, and parental behavior were outlined as particular concerns. In terms of processing and managing VAHCW, we also identified decision dilemmas and the personality traits of employees as risk factors (Moylan and Cullinan, 2011).

Infrequent yet distinctly Hungarian-specific elements, such as the perception of cultural conflict, were present. Our sample uncovered biased views about the Romani minority in such conflicts. We found no reference to this in any literature. The cultural conflicts between the relatives of inpatient children and staff members cause daily operational difficulties because they jeopardize the process of visiting. This is influenced by a larger societal challenge in Hungary, which highlights the lack of empathy toward groups with lower SES and lower educational backgrounds (Pich et al., 2011). This finding is important because it is unlikely that the phenomenon would have been expressed through direct questioning. Furthermore, 71% of the respondents were female, yet violence against women appeared in only four items (19%), in the themes of physical, and non-contact abuse, and case descriptions, again somewhat contradicting previous research findings (Gale et al., 2009). One of the most frequent features of non-physical violence was bullying, which occurred in both face-to-face and online spaces. In describing incidents, the child’s antisocial traits were more often mentioned than psychiatric causes, i.e., respondents had different thoughts about bullying in general and when they recalled it. When recollected, the child’s antisocial personality development is more prominent, while the psychiatric pathology is referred to the background. Broader societal causes are also highlighted, as well as the fact that the Hospital alone cannot resolve the situation. This suggests that also in the view of the employees these problems are rooted in wider social anomalies.

It should be emphasized that the generalizability of the results is limited due to the study’s focus on a single specific psychiatric ward. Furthermore, the surveyed group of individuals was relatively small in size. Additionally, the diverse range of professions makes comparisons challenging.

## Discussion

This research is limited to the experiences of the staff within our Unit. Our facility is small, so the response and its content may have been distorted by group dynamics. From this perspective, our findings can only provide a starting point for exploring the nature of violence against staff in child psychiatry. Due to the sensitivity of the topic and ethical considerations, we did not investigate bullying among the staff. For these delicate reasons, no audio recording was used, the data set was generated by typing. We consider that our results may allow us to ask valid questions about VAHCW in a future survey on a larger sample of other psychiatric wards or other childcare settings, and to assess the social structure and criminological and psychiatry trends behind the abuse.

If a child or a parent threatens healthcare staff, it may amount to violence against a public official, which can be prosecuted in Hungary even in some cases for perpetrators under the age of 14. In such cases, the Hospital will press charges. Our study shows that healthcare staff perceives the threat as the most common non-contact violence. Therefore, the threat should be taken seriously and reported in all cases.

The extent to which a psychiatric disorder influences the actions of perpetrators is a legal and forensic matter. Our research provides examples, such as throwing a chair or inflicting a punch resulting in a broken upper arm, which can be interpreted as criminal acts with potential imprisonment sanctions. The presence of CCTV surveillance at the ward entrance serves as a deterrent and holds legal implications. Additionally, actions like tearing clothes and spitting may be considered defamation or even assault under Hungarian legislation.

On the contrary, from a human rights perspective, it is essential to acknowledge the issue of mechanical restraint and other forms of restrictive measures. According to the 16th Annual Report of the CPT^1^ (URL1), paragraph 54, the frequency of use of restraint measures can have a detrimental effect on the workplace environment and should be avoided wherever possible, but paragraph 37 declares that in some situations the safety of the patient and the staff is also important. Our research obviously demonstrated that in such a situation the decision dilemma may be a risk factor.

From a practical standpoint, our research highlights the likelihood that women in healthcare refrain from explicitly addressing the specific nature of the abuse they encounter, despite being well-aware of its existence. Children at earlier stage of antisocial personality development appear relatively more frequently at our ward, as previous research also has implicated (Perry, 1984; Neumann and Klatt, 2022). These adolescents, mostly boys, are confronted by female nurses during their treatment in the ward. As the majority of healthcare workers are women, VAHCW may be a specific form of violence against women (Gale et al., 2009). The practical experience that supports our results is that hetero-anamnestic data on children are mostly obtained from mothers, which may suggest that in families with a patriarchal structure, the mother plays the traditional caregiver role while the father is responsible for family maintenance. The latter may include both protection and domestic violence. In families organized based on non-traditional roles, women also work, but fathers in patriarchal cultural settings can be as aggressive with female employees as with homemaker women in their families. This draws our attention to the specific Hungarian gender inequalities and to the fossils of medieval social structures.

In our pilot study, we have identified several significant risk factors that are highly likely to provoke VAHCW. These are the admission, mechanical restraint, medication withdrawal (the children did not take their prescribed medication for some reason), visiting, and failures of patient-staff communication. All five situations are related to some form of interaction. In our opinion, social skill trainings can solve these shortcomings exclusively with external experts in all child psychiatric wards. Further education and debriefing seem to be important. Mindfulness can be a key to the prevention of violence against healthcare workers (Brunero and Stein-Parbury, 2008; Bryant, 2010). Brewer (1999) indicated that assertiveness is crucial in the immediate prevention of violence and that confidentiality must not compromise personal safety. A qualitative study conducted in Canadian child psychiatric hospitals (Faulkner-Gibson, 2012) found that interpersonal relationships between colleagues influence the perceptions of children’s aggressive behavior. The perception of aggression lies on a continuum that is triggered by the group dynamics among staff members. The integration of individual and group clinical supervision into the work schedule can reduce burnout and moral distress. Discussing and sharing practice issues (debriefing) reduces anxiety, burnout, and the frequency of conflicts between colleagues. Effective focus on the situation, self-awareness, self-confidence, good time management, regular feedback, and the development of communication techniques are crucial. It is imperative to emphasize that individuals entering the psychiatric field should have a profound understanding of their fears, anxieties, and sensitivities, as emphasized by Meerwijk et al. (2007). While the findings are supported, further intensive research is necessary to strengthen their credibility. Extending the material would contribute to a more robust understanding of the subject.

## Data availability statement

The raw data supporting the conclusions of this article will be made available by the authors, without undue reservation.

## Ethics statement

The studies involving humans were approved by Heim Pal National Pediatric Institute IKEB (Hungarian acronym for Institutional Board of Research Ethics) (Authorization number: KUT-26/2022). The studies were conducted in accordance with the local legislation and institutional requirements. The participants provided their written informed consent to participate in this study.

## Author contributions

GS: conceptualization, data curation, and supervision. KT: validation. GC: validation, and translation. GF: formal analysis, investigation, methodology, project administration, translation, writing—original draft, and writing—editing. All authors contributed to the article and approved the submitted version.

## Footnotes

^1^European Committee for the Prevention of Torture and Inhuman or Degrading Treatment or Punishment (CPT).
